# Subjective Well-being of Special Education Teachers in China: The Relation of Social Support and Self-Efficacy

**DOI:** 10.3389/fpsyg.2022.802811

**Published:** 2022-02-15

**Authors:** Wangqian Fu, Lihong Wang, Xiaohan He, Huixing Chen, Jiping He

**Affiliations:** ^1^School of Special Education, Beijing Normal University, Beijing, China; ^2^Beijing NO. 8 High School, Beijing, China; ^3^School of Education, University of Malaya, Kuala Lumpur, Malaysia; ^4^Guangzhou Qicong School, Guangzhou, China

**Keywords:** special education teachers, social support, self-efficacy, subjective well-being, China

## Abstract

In order to explore the relationship of social support, self-efficacy, and subjective well-being of special education teachers in China, 496 teachers from 67 special education schools were surveyed by questionnaire. We found that (1) the subjective well-being of special education teachers in China was in the medial level. (2) There were significant differences in subjective well-being level among teachers of different genders, teacher position, education background, and teaching age. Male teachers were of higher subjective well-being; subjective well-being of head teachers was lower than those were not head teachers; teachers with the educational background of postgraduate were of higher relaxation and tension than those with junior college educational background; the control scores of emotion and behavior of teachers with teaching age of 3 years and below were significantly lower than those of teachers with teaching age of more than 10 years. (3) Self-efficacy played a partially mediating role in the relationship between social support and subjective well-being of special education teachers. Suggestions to improve the subjective well-being of special education teachers were discussed in the article.

## Introduction

Since the end of the 20th century, after Seligman proposed positive psychology, psychologists have begun to look at the potential, motivation, and abilities of people with a more open and appreciative attitude (Seligman and Csikszentmihalyi, 2000; Sheldon and King, 2001). As one of the important contents of positive psychology, subjective well-being (SWB) has attracted the attention of scholars all over the world. SWB benefits individuals in many aspects such as health and longevity, work, income, and friendship (Diener and Ryan, 2009; Diener and Chan, 2011). SWB was the overall feelings and judgments of an individual about their quality of life, which was based on their subjective standards. It has the characteristics of subjectivity, stability, integrity, etc. Its components include satisfaction with personal life and important fields in life, positive emotional experience and low-level negative emotional experience (Diener et al., 1999).

### Impact of Subjective Well-Being of Teachers for Teachers and Students

For teachers, the level of SWB reflects their mental health and reflects their satisfaction with their current life and work conditions. SWB would not only affect their work enthusiasm and initiative (Du, 2017; Buriæ et al., 2019), but also affect the mental health of students (Liu, 2014; Perera et al., 2019). Teachers with lower levels of mental health usually behave negatively and passively when facing challenges in teaching (Bi, 2013). A survey showed that special education teachers have more severe mental health problems. Among them, teachers with mild or above psychological problems account for 25% of the total (Zhao and Wang, 2013). Compared with general teachers, special education teachers, due to the particularity of their education targets, lack a sense of accomplishment by working much with low effectiveness, which leads to a low happiness index (Wan and Zhao, 2013). They are more likely to experience a state of low happiness earlier (Shen et al., 2012). With the successive promulgation of policy documents such as the “Opinions on Comprehensively Deepening the Reform of Teacher Team Building in the New Era,” “Opinions on Strengthening the Building of Special Education Teacher Team,” “Special Education Teacher Professional Standards (Trial),” and other policy documents by the State Council and the Ministry of Education, more specific requirements for the professional development of education teachers have been made. It has been further pointed out that teachers should be the instructors of the healthy growth of students. The opinions also mentioned that the benefits and rights protection should be strengthened to further expand the happiness of teachers (The Central Committee of the Communist Party of China and the State Council, 2018). For the special education teacher group in China, improving their (happiness) development and exploring their key influencing factors and their mechanism of actions are urgent (Peng et al., 2018).

### Relationship Between Social Support and Subjective Well-Being of Teachers

Scholars have found that the social support is one of the important predictors of SWB (Meehan et al., 1993; Song and Fan, 2013). From the perspective of the main components of it, the social support can be divided into the informal and formal social support. The former mainly refers to the support and assistance provided by informal social relations resources such as family, neighbors, friends, and colleagues; the latter refers to the guarantee and support provided to people through formal organizations such as government, enterprises, community organizations, and formal institutional arrangements (Zhang, 2001). The social support can also be divided into objective support and subjective support as well. That is, the actual support that can be seen and the emotional support that is experienced (Langford et al., 1997; Langher et al., 2017). Good social support has a clear positive correlation with life satisfaction, a significant positive correlation with positive emotions, and a clear negative correlation with negative emotions, including the loneliness, depression, and anxiety (Lam, 2019; Gkda, 2021). The higher the degree of social support, the stronger the SWB (Lu and Ying, 2014; Lam, 2019). González and Restrepo-Chavarriaga (2010) found that there was a close connection between social support and well-being: the higher the level of social support, the higher the quality of life of people. People who have good social support, good interpersonal communication and relationships, and the more positive emotions they experience, the higher the degree of happiness they would feel (Ding, 2007). Numerous studies find the positive association between social support and well-being of teachers in global context, including United States (Carroll, 2020), Macao (Huang, 2011), Romanian (Stanculescu, 2014), and so on.

### Role of Self-Efficacy Between Social Support and Subjective Well-Being of Teachers

How does social support of teachers affect their SWB? The self-efficacy may be an important mediating factor, which refers to the subjective judgment of people on whether they can successfully perform a certain behavior for achievements (Bandura, 1977). The social support may affect the inner psychology of the individual or it may affect the psychological quality of the individual by affecting some of the inner psychological factors of the individual (Uchino et al., 1996). The effect of external social factors on the inner psychology of an individual needs to go through the mediating effect of self-concept (Judge et al., 1997) and self-efficacy is one of the important components of self-concept (Liu, 2001). Bandura (2008) believed that the self-efficacy, optimism, hope, and resilience played an important role in managing and coping with stress and improving happiness. Studies have pointed out that there is a significant correlation between self-efficacy and SWB (Caprara and Steca, 2005; Yu et al., 2005). For teachers, the higher the self-efficacy they feel, the higher the level of SWB there is (Cui, 2016). When teachers possess high self-efficacy to perform required tasks (Klassen and Chiu, 2011), they would experience favorable outcomes, including satisfaction (Zee and Koomen, 2016) and well-being (Huang et al., 2019; Perera et al., 2019; Moè and Katz, 2020). Studies have shown that social support can help individuals to improve their coping ability and improve their sense of efficacy in their own coping capabilities, thereby reducing negative reactions to stressful events and promoting individual growth (Cohen and Syme, 1985; Stanculescu, 2014). When teachers have high level of perceived social support and a good sense of their abilities to affect the outcomes of students (teacher self-efficacy), they experience SWB.

### Current Study

Overall, previous studies focus on SWB of teachers (Chan, 2013; Renshaw et al., 2015; Farhah et al., 2021), while SWB of special education teachers was neglected. Besides, few previous studies explored and proved mediating effect of self-efficacy between the social support and their SWB of special education teachers in China. A deeper reciprocal relation between the relation in social support, efficacy and SWB of special education teachers should be investigated in China. To fill in the gaps, we investigated SWB of special education teachers in China and mediating reaction was explored on the mediating effect of self-efficacy between social support and their SWB of special education teachers. Drawing on prior empirical findings and self-efficacy theory, we tested the following hypotheses: (1) social support of special education teachers was positively associated with their SWB in China; (2) self-efficacy of special education teachers was positively associated with their SWB in China; and (3) self-efficacy of special education teachers played the mediating role between social support and SWB of teachers.

## Materials and Methods

### Participants and Procedure

We contacted the principals of special education schools to help send the questionnaire link to special education teachers. There were 508 special education teachers in 67 special education schools participating in this study. After deleting the questionnaires missing one-third questions and with all the same choices in the items, eventually 496 valid questionnaires were collected, with 97.6% recovery rate. The basic information of the research sample was: there were 108 male teachers (21.8%) and 388 female teachers (78.2%); 441 Han teachers (88.9%) and 55 ethnic minority teachers (11.1%); most teachers have educational backgrounds in the undergraduate and junior college (408 undergraduate teachers and 72 junior college teachers, a total of 96.8%), including 197 teachers with special education majors (39.7%) and 276 teachers with non-special education majors (55.6%) and there were 23 teachers with non-educational majors; from the perspective of teaching experience, there were 110 teachers with 3 years or less of teaching experience (22.2%), 62 teachers with 3–5 years of teaching experience, 81 teachers with 6–10 years of teaching experience, and 243 teachers with more than 10 years of teaching experience (49%). Of all the 496 teachers, a total of 280 teachers were head teachers and the remaining 216 teachers were not head teachers.

### Measures

#### Social Support Scale

The Social Support Scale compiled by Xiao in 1990 was adopted in this study. The scale has been proven to have good reliability and validity through a large number of studies (Liu et al., 2013). There were 10 items in total in this scale, including three dimensions: the objective support (sum of scores in items 2, 6, and 7, e.g., what were the sources of financial and tangible supports you have received when you were experiencing difficult emergencies), the subjective support (sum of scores in items 1, 3, 4, and 5, e.g., how many close friends that can you ask for help and support), and the utilization of support (sum of scores in items 8, 9, and 10, e.g., who would you turn to when you were in trouble). The total score of social support was the account of the scores in all the ten items. The internal consistency reliability coefficient of the scale in this study was 0.76.

#### Subjective Well-Being Scale

The SWB Scale revised by Duan in 1996 was used in this study. The original scale was a standardized test tool developed by the National Center for Health Statistics to evaluate the statements of happiness of subjects. It consisted of 33 items. The higher the score, the stronger the happiness. The first 18 items of the scale were used by Duan to measure the subjects and the correlation between each item of the scale and the total score was between 0.48 and 0.78 (Fazio, 1977). The correlation between the individual item score and the total score was between 0.49 and 0.78; the correlation between the subscale and the total scale was between 0.56 and 0.88; the internal consistency coefficient was 0.91 for males, 0.95 for females, and the test-retest reliability was 0.85. Thus, the validity index was considered valid. The scale was constituted by 6 factors: the worry about health [e.g., have you been bothered by illness, discomfort, pain, or fear of illness (in the past month)], energy [e.g., did you wake up feeling refreshed and energized (in the past month)], satisfaction and interest in life [e.g., how happy, fulfilled, or enjoyable your life has been (in the past month)], melancholy or happy mood [e.g., have you ever wondered if anything was worth doing because you were sad, discouraged, disappointed, or in a lot of trouble (in the past month)], control of emotion and behavior [e.g., have you been firmly in control of your actions, thoughts, emotions, or feelings (in the past month)], and relaxation and tension (anxiety) [e.g., are you under or have you been under any restraint, stimulation, or pressure (in the past month)] (Duan, 1996). The reliability coefficient of internal consistency in this study was 0.89.

#### Teachers’ Self-Efficacy Scale

The Teachers’ Self-efficacy Scale was compiled by Tshannen-Moran and Hoy (2001) and was revised in Chinese by Lu (2017). A total of 24 items were included in the scale. The self-efficacy of the teacher was evaluated from three different dimensions, including the student management (e.g., you feel how much you can do when you help your students to think deeply), instructional strategy (e.g., how well have you done adapting the course to the level of the students), and class management (e.g., how good are you at keeping a few problem students from ruining the class?). The Likert 9-point scale was applied in the scale for scoring. The higher the score, the higher the sense of self-efficacy (Dixon et al., 2014). In this study, the internal consistency coefficient of Chinese version of the scale revised by Lu (2017) reached 0.95. The internal consistency coefficients of the three dimensions of the student engagement, teaching strategy, and classroom management were 0.875, 0.876, and 0.881, respectively, and the test-retest reliability was 0.76, 0.80, and 0.85, respectively. Therefore, the research of this study should be reliable. The content and structure of the scale were reasonable that found by the content validity and structure validity test as well (Lu, 2017). The internal consistency reliability coefficient of the scale in this study was 0.95.

## Data Analysis

Differences in SWB levels among teachers by their sociodemographic variables were tested with ANOVAs and *t*-tests in SPSS software version 20.0 (SPSS Incorporation, Chicago, IL, United States). *Post hoc* analyses were subsequently performed by least significant difference (LSD). Second, the correlation of SWB, social support, and self-efficacy were analyzed using the Pearson’s correlation coefficient function in SPSS. Third, to test whether self-efficacy plays a mediating role between social support and SWB, the mediation model was applied using the PROCESS macro in SPSS.

## Results

### Subjective Well-Being of Special Education Teachers in China

Subjective well-being status of special education teachers in China is shown in Table 1. Overall SWB level of special education teachers in China scored 74, among which female teachers scored 72 and male teachers scored 78. SWB score of special education teachers in China was at a medial level among teachers, since the average score of elementary education teachers was 73.2 (Xin et al., 2021).

**TABLE 1:** The basic situation of subjective well-being of special education teachers in China.

Total score and dimensions	*M*	*SD*	*Max*	*Min*
Total subjective well-being score	73.57	13.69	109	20
Sub-concern about health	6.92	2.47	16	1
Sub-energy	17.59	4.33	27	4
Sub-satisfaction and interest in life	6.38	1.85	11	2
Sub-depression or happy mood	15.23	3.51	22	2
Sub-control of emotion and behavior	12.47	2.21	17	3
Sub-relaxation and tension (anxiety)	14.98	4.08	26	3

The difference of SWB of special education teachers in China was analyzed on different background variables (see Table 2). In terms of education background, teachers with different education levels differed significantly on the dimension of relaxation and tension (*F* = 4.237, *p* = 0.015 < 0.05) (see Table 3), but not for total SWB and other dimensions. The teachers of bachelor degree were significantly lower than those graduated from junior college (*P* = 0.024 < 0.05) and postgraduate or above (*P* = 0.048 < 0.05). Two were on the dimension of relaxation and tension. There were no significant differences in SWB levels among teachers with different teaching years, while multiple tests showed that teachers with 3 and fewer years of teaching experience had significantly lower scores control of emotion and behavior than teachers with more than 10 years of teaching experience (*F* = 0.52, *p* < 0.05). There were significant differences in total SWB scores of teachers by genders. SWB of male special education teachers was much higher than female ones (*F* = 3.754, *p* < 0.001), specifically on four dimensions of energy factor (*F* = 3.725, *p* < 0.001), including satisfaction and interest in life (*F* = 3.12, *p* < 0.01), depressed or happy mood (*F* = 2.695, *p* < 0.01), and control of emotions and behaviors (*F* = 3.778, *p* < 0.001). There were significant differences in dimensions of SWB (*F* = −1.98, *p* < 0.05) and the subdimension control of emotions and behaviors (*F* = −2.08, *p* < 0.05) for special education teachers whether they were head teachers.

**TABLE 2:** The differences by education, teaching experience, gender, and whether they were classroom teachers on subjective well-being.

		Total score and dimensions (M ± SD)	Sub-concern about health (M ± SD)	Sub-energy (M ± SD)	Sub-satisfaction and interest in life (M ± SD)	Sub-depression or happy mood (M ± SD)	Sub-control of emotion and behavior(M ± SD)	Sub-relaxation and tension (anxiety) (M ± SD)
Educational background	Junior College	75.97 ± 13.23	6.60 ± 1.92	18.19 ± 3.98	6.43 ± 1.72	15.99 ± 3.78	12.83 ± 2.11	15.93 ± 4.38
	Undergraduate	73.01 ± 13.73	6.97 ± 2.55	17.45 ± 4.39	6.38 ± 1.87	15.08 ± 3.48	12.38 ± 2.22	14.75 ± 3.96
	Postgraduate or above	77.40 ± 14.38	6.93 ± 2.40	18.67 ± 4.20	6.07 ± 1.91	15.40 ± 2.87	13.47 ± 2.23	16.87 ± 4.98
F		2.037	0.710	1.393	0.241	2.050	2.849	4.237**
P		0.131	0.492	0.249	0.786	0.130	0.059	0.015
Teaching experience	below 3 years	73.49 ± 13.56	7.14 ± 2.5	17.61 ± 4.18	6.42 ± 2.01	15.15 ± 3.44	12.21 ± 2.30	14.96 ± 4.19
	3-5 years	73.82 ± 15.19	7.18 ± 2.8	17.24 ± 4.66	6.37 ± 1.95	15.39 ± 3.8	12.15 ± 2.14	15.50 ± 4.60
	6-10 years	72.53 ± 12.15	6.80 ± 2.24	17.43 ± 3,91	6.22 ± 1.65	15.01 ± 2.86	12.33 ± 2.06	14.73 ± 3.43
	10 above	73.89 ± 13.91	6.80 ± 2.44	17.72 ± 4.45	6.41 ± 1.81	15.29 ± 3.67	12.72 ± 2.22	14.94 ± 4.11
F		0.207	0.765	0.247	0.229	0.187	2.142	0.444
P		0.892	0.514	0.863	0.876	0.905	0.094	0.722
Gender	Male	77.89 ± 12.19	7.22 ± 2.59	18.94 ± 4.24	6.86 ± 2	16.03 ± 3.75	13.18 ± 2.09	15.66 ± 3.89
	Female	72.37 ± 13.86	6.84 ± 2.43	17.21 ± 4.28	6.24 ± 1.78	15.01 ± 3.41	12.28 ± 2.21	14.79 ± 4.12
T		3.754***	1.436	3.725***	3.12**	2.695**	3.778***	1.95
P		0.000	0.152	0.000	0.002	0.007	0.000	0.052
Being head teachers in classroom	Yes	72.50 ± 13.15	6.82 ± 2.28	17.27 ± 4.26	6.30 ± 1.76	15.03 ± 3.34	12.29 ± 2.16	14.80 ± 3.98
	No	74.95 ± 14.28	7.06 ± 2.69	18 ± 4.39	6.47 ± 1.96	15.49 ± 3.71	12.71 ± 2.26	15.22 ± 4.21
T		−1.98*	−1.07	−1.88	−1.03	−1.47	−2.08*	−1.15
P		0.048	0.287	0.061	0.304	0.143	0.038	0.250

*p<0.05, **p<0.01, ***p<0.001.

**TABLE 3:** Multiple comparisons (LSD).

Dependent Variable	(I) Educational background	(J) Educational background	Mean Difference (I-J)	Std. Error	Sig.
Sub-relaxation and tension (anxiety)	Junior College	Undergraduate	1.178*	0.519	0.024
		Postgraduate or above	–0.936	1.152	0.417
	Undergraduate	Junior College	−1.178*	0.519	0.024
		Postgraduate or above	−2.114*	1.067	0.048
	Postgraduate or above	Junior College	0.936	1.152	0.417
		Undergraduate	2.114*	1.067	0.048

*p < 0.05.

### Correlation of Social Support, Subjective Well-Being, and Self-Efficacy

The Pearson correlation was used to analyze the correlation among the social support, SWB, and self-efficacy of special education teachers (see Table 4). There were significant positive correlation between social support and its three subdimensions and SWB and the three dimensions of social support, objective support, subjective support, and utilization of support were significantly and positively correlated with the energy factor of SWB, satisfaction and interest in life, depressed or happy mood, control over emotions and behavior, and relaxation or tension. Meanwhile, there were significant positive correlation between the social support and its three dimensions and self-efficacy and its dimensions, which were significantly and positively correlated with self-efficacy and its three dimensions. Besides, there were significant positive correlation between self-efficacy and its three dimensions and SWB.

**TABLE 4:** The correlation of teachers’ social support, subjective well-being and self-efficacy (*n* = 496).

	1	2	3	4	5	6	7	8	9	10	11	12	13	14
1 Total subjective well-being														
2 Sub-concern about health	0.320**	1												
3 Sub-energy	0.855**	0.079	1											
4 Sub-satisfaction and interest in life	0.656**	–0.02	594**	1										
5 Sub-depression or happy mood	0.883**	0.123**	0.752**	0.536**	1									
6 Sub-control of emotion and behavior	0.668**	0.082	0.478**	0.460**	0.536**	1								
7 Sub-relaxation and tension (anxiety)	0.838**	0.245**	0.585**	0.419**	0.696**	0.473**	1							
8 self-efficacy	0.334**	−0.220**	0.336**	0.281**	0.373**	0.345**	0.264**	1						
9 Sub-student engagement	0.342**	−0.207**	0.347**	0.311**	0.380**	0.315**	0.267**	0.940**	1					
10 Sub-teaching strategy	0.306**	−0.210**	0.324**	0.257**	0.335**	0.313**	0.236**	0.959**	0.845**	1				
11 Sub-classroom management	0.305**	−0.212**	0.324**	0.233**	0.347**	0.357**	0.249**	0.955**	0.831**	0.897**	1			
12 Total social support	0.385**	0.059	0.304**	0.282**	0.371**	0.306**	0.321**	0.329**	0.307**	0.311**	0.321**	1		
13 Sub-objective support	0.345**	0.066	0.255**	0.262**	0.329**	0.291**	0.288**	0.259**	0.230**	0.245**	0.266**	0.865**	1	
14 Sub-subjective support	0.279**	0.045	0.232**	0.199**	0.261**	0.228**	0.223**	0.269**	0.258**	0.254**	0.254**	0.823**	0.519**	1
15 Sub-utilization of support	0.258**	0.009	0.217**	0.179**	0.269**	0.158**	0.233**	0.238**	0.234**	0.224**	0.220**	0.550**	0.320**	0.251**

*p<0.05, **p<0.01, ***p<0.001.

### Mediation of Self-Efficacy Between Social Support and Subjective Well-Being

**FIGURE 1:**
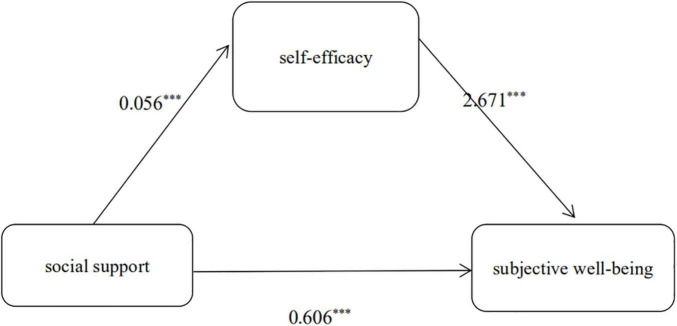
The mediated effect model of social-efficacy between social support and subjective well-being of special education teachers in China. ^***^p < 0.001.

We test whether self-efficacy played a mediating role in the relationship between social support and SWB. The independent variables (social support), dependent variables (SWB of teachers), and intermediary variables (self-efficacy) are included in the model under the control of gender, teaching age, and professional background of teachers and tested by bootstrap. The results are shown in Table 5 and Figure 1. As shown in Table 5, the three paths of the social support and self-efficacy, the self-efficacy and SWB, and the social support and SWB were all extremely significant. Social support had direct effect on SWB and also to increase SWB of teachers by positively associated with their self-efficacy suggested that self-efficacy has a partial mediating effect between the social support and SWB. The direct effect of social support to SWB was 0.606, the indirect effect of social support to SWB was 0.149, and the total effect of social support to SWB was 0.755.

**TABLE 5:** The mediation effects of self-efficacy (2000 Bootsraps).

independent variable	dependent variable	Mediate variables	X→M		M→Y		X→Y		direct effect		indirect effect		total effect	
			coeff	SE	coeff	se	Coeff	Se	coeff	se	coeff	95%CI	coeff	se
social support	subjective well-being	self-efficacy	0.056***	0.004	2.671***	0.245	0.606***	0.081	0.606***	0.081	0.149***	0.086,0.223	0.755***	0.08

X is social support, M is self-efficacy, Y is subjective well-being. *p<0.05, **p<0.01, ***p<0.001

## Discussion

### Subjective Well-Being of Special Education Teachers in China

The results of the survey on SWB of the special education teachers in China show that their SWB was on the average level of Chinese elementary schools teachers (Xin et al., 2021). It is in line with finding of Kyriacou (2011) and is a little different with the findings of Huang et al. (2016) and Olagunju et al. (2020), pointing out that SWB of special education teachers is low. Kyriacou (2011) found that, despite teaching being recognized as a high-stress occupation, teachers are reportedly healthier than other the professional groups. While for special education teachers, Olagunju et al. (2020) found that about four in every ten Nigerian special education teachers had psychological distress. The education system and culture have great impact in SWB of teachers (Xin et al., 2021). The reason of difference with Huang et al. (2016) may be that the survey groups are located in different regions of China. Besides, the government has paid more and more attention to the development of special education, issued a series of policies to support the construction of special education schools, and supported the construction of special education teachers by improving the treatment of special education teachers and promoting their professional development (Zhang and Wei, 2014). The special “national training plan” has been carried out in rural areas, which could promote SWB of special teachers in China to some extent.

By analyzing the demographic variables that impact SWB of special education teachers, it was found that SWB level of teachers was associated with many factors, including gender and teaching age, whether being a head teacher. There are significant differences in SWB level of special education teachers of different genders. Male special education teachers in northwest China had higher SWB and the scores of the total score of happiness, energy factor, satisfaction and interest in life, melancholy or happy mood, and control of emotion and behavior are significantly higher than those of female ones. The association between gender of SWB teachers remains controversial. Some studies based on teacher gender theory have pointed out that female teachers are generally significantly higher than male ones in terms of happiness level (Liu, 2011; Wang, 2017), while some research found that female teachers reported a higher level of psychological distress (Pevalin, 2000; Dai, 2014; Chaplain and Jackson, 2017). The reason may be as female teachers, since they not only have to face the same educational and teaching pressure as male ones, but also have to bear more responsibilities of family such as bearing, raising, educating children, and housework in China (Ding, 2006), which may result that the happiness of female special education teachers in China is significantly lower than that of male ones.

In addition, professional-related factors of teachers, including education background, teaching years, and whether they are head teachers, all have association with SWB level of special education teachers. The results showed that there was no significant difference in the total score of SWB, but in the dimension of relaxation and tension among the special education teachers with different educational backgrounds in China. The teachers of bachelor degree were significantly lower than others in the dimension of relaxation and tension. On one hand, teachers of bachelor degree felt more anxious than those with higher education background. Teachers graduated from master program are more confident in the teaching work and be more trusted by the administrators in the school (Liang, 2017) and they are also less likely to suffer from anxiety (Wang et al., 2009). On the other hand, teachers of bachelor degree felt more anxious than those graduated from junior college. It is similar with study of Zhang (2020). She found that special education teachers graduated from junior college scored higher in job satisfaction than undergraduate teachers. It is since teachers graduated from junior college have low expectation on their career and are relatively less under pressure in the work (Wang, 2017).

In terms of teaching age, the teachers with more than 10 years of teaching experience scored significantly higher than ones with 3 years or less on the emotion and behavior control dimension of SWB, which indicated that the work pressure of teachers with longer teaching experience was significantly lower than that of ones with shorter teaching experience (Pang, 2003) and they have higher sense of self-efficacy (Vasher, 2012) and SWB. Teachers who have longer teaching experience will more consciously conduct self-education, self-perfection, and self-improvement in teaching management (Gavish and Friedman, 2010; Aloe et al., 2014; Ren and Wang, 2015), whereas teachers who are new and have shorter experience are more likely to suffer from stress in teaching management, time management, and work-life balance. Without proper social support, they are 60% less likely to stay in teaching for the long term (Calnan, 2017).

This study result showed that SWB level of special education teachers serving as head teachers was significantly lower than that of ones not in the northwest region of China. There are significant differences on the control of the emotional and behavioral dimensions; the teacher who do not work as a head teacher can better control of their emotions and behavior. Consistent with previous studies (Dai, 2014; Chaplain and Jackson, 2017), i.e., positions of teachers have a significant impact on their level of happiness, the teacher incharge of the class is usually affected by the school chores and heavy workload, so they are faced with greater work pressure as well as a lower sense of self-efficacy. Dramatic changes to the role of a modern head teacher have been associated with increased levels of stress (Chaplain and Jackson, 2017). For special education head teachers, they are responsible for organizing the daily running of the classroom and dealing with challenge behaviors of students (Langher et al., 2017), whose SWB is low. Because of students with disabilities have great individual differences, generally weak learning ability and most accompanied by more emotional and behavioral problems, they also need to deal with the curriculum organization and management and solve unexpected problems.

### Mediation Effect of Self-Efficacy on the Relationship Between Social Support and SWB of Special Education Teachers

The results showed that the level of social support obtained by special education teachers in China can positively predict their SWB level. That is, the higher the social support level, the SWB level will be higher. It is consistent with the previous research (Lam, 2019). Social support is closely related with SWB of teachers (Lam, 2019; Carroll, 2020). The study of Chinese researchers also shows that different dimensions of social support of special education teachers have a good predictive effect on SWB of teachers and its various dimensions (Tang, 2006; Lu and Ying, 2014). Social objective support can provide material or information help, increase happiness of teachers, and improve their self-confidence and the increase of utilization of support of an individual will reduce worries and the possibility of their inner depression and pain. Therefore, teachers with high utilization of social support usually have a higher level of SWB (Zhang, 2005; Chaplain and Jackson, 2017; Langher et al., 2017) and the special education teachers who can make good use of various social support resources usually feel less psychological pressure, have more positive task motivation, and then have higher SWB (Wang et al., 2012).

Further analysis found that self-efficacy of teachers played a mediating role in the relationship between social support and SWB of special education teachers in northwest China. That is, social support affected SWB of teachers level by improving self-efficacy of teachers. Social support not only directly affects SWB of special education teachers, but also impacts on self-efficacy and improves their SWB of teachers (Huang, 2011; Stanculescu, 2014; Carroll, 2020). On one hand, social support can have a direct effect through encouragement. On the other hand, it can also have an indirect effect through changing self-efficacy, so as to change individual behavior and produce a lasting effect on such change (Jiang et al., 2004). Meanwhile, the higher the level of social support, the more likely to promote individuals to adopt positive coping strategies to solve problems (Chan, 2002; Watts, 2014). Teacher needs personal accomplishment serve as emotional resources that favors positive personal outcomes (Katz and Shahar, 2015; Aelterman et al., 2016). As a high-order personality structure, the sense of self-efficacy is closely related with the psychological state of an individual: the higher the level of the sense of self-efficacy of an individual, the higher the level of SWB (Huang et al., 2012). High social support gives confidence of special education teachers in their own teaching ability and makes them have higher evaluation of life quality as well as SWB.

## Limitation and Further Research

Limitations in this study were noted. First, the main limitation of this study is its cross-sectional nature that does not allow to infer causal relationships between the studied variables. Further studies can be elucidating the complexity of the reciprocal relations between these variables by examining the role of potential mediators longitudinally. Second, although the study focus on SWB of Chinese special education teachers, the comparison of special education teachers in a broader context would be help to understanding the impact of culture. An important area of future research will be conducted in different countries.

## Practical Implication

First, for the development of special education in different regions, the government should set quantifiable and actionable policy goals. In this study, the research on SWB of special education teachers is inconsistent with previous studies in central and eastern regions, which main reason lies in the unbalanced development of special education in different regions making different working environment of special education teachers. “The Opinions of the General Office of the State Council on Further Adjusting and Optimizing the Structure to Improve the Efficiency of the Use of Educational Funds” points those educational resources should be tilted to remote and poor areas to promote the rational allocation of educational resources and educational equity. All the relevant policy documents, such as “the First and Second Special Education Promotion Plan and Opinions on Comprehensively Deepening the Reform of Teacher Team Construction in the New Era”, indicate that the construction of special education teachers has become a key task for the development of special education at this stage. However, there are some deviations in the implementation of the policy and it is necessary to formulate operational and instructive local policies to promote the development of regional special education and to procedurally supervise the implementation of the policies.

Second, school organizations should provide targeted support to teachers of different genders and teaching ages. Any kind of behavior or psychological expression arises from the totality of various interdependent facts, which have the characteristics of a dynamic field. School organization is one of the most leading social environments for teachers, which has a direct impact on the quality of life of teachers. This study shows that female and newly recruited special education teachers in China have a low SWB, which can be effectively elevated by improving their social support and self-efficacy. Therefore, school organizations should fully respect their work and give certain social support, including psychological counseling, flexible teaching adjustment during lactation period, carrying out colorful activities, and so on, to show the charm of female teachers and enhance their self-confidence. In fact, new teachers often lack a clear plan for their future career development. They are prone to various problems in teaching and feel great pressure. They need to improve their sense of self-efficacy by elevating their professional ability. Schools can help new teachers to adapt to the teaching environment as soon as possible and improve their teaching skills through class evaluation, teaching on behalf of teachers as well as teaching and research groups. At the same time, we should make full use of national and provincial training programs to provide relevant professional training opportunities for young special education teachers to meet their professional development needs.

Third, the on-service training system for special education teachers in China should be improved. The establishment and improvement of the social support system needs the joint efforts of the school, society, and teachers. “What Every Special Educator Must Know: Ethics, Standards, and Guidelines for Special Educators,” emended by the Council for Exceptional Children (2022), which stipulates that special education teachers are required to develop a career plan during their post-service development and have the right to seek adequate professional development resources from their institutions. Social resources are of great significance to the professional development of special education teachers and the improvement of their quality of life. The government and schools should provide effective training opportunities and sufficient training resources for teachers, effectively improve their sense of teaching efficacy, enable them to correctly deal with the possible new situations and situations in the working situation, and adapt to the new work objects as well as contents more quickly. In addition, scientific teaching evaluation mechanism is the quality assurance of social support system operation. Scientific teaching evaluation mechanism cannot only promote career planning and work effectiveness of special education teachers, but also enhance their social support utilization degree, so as to improve SWB of this group and promote the improvement of special education quality.

## Data Availability Statement

The original contributions presented in the study are included in the article/supplementary material, further inquiries can be directed to the corresponding author/s.

## Ethics Statement

The studies involving human participants were reviewed and approved by Beijing Normal University. The patients/participants provided their written informed consent to participate in this study.

## Author Contributions

WF designed the study, collected the data, and wrote the manuscript. LW collected and analyzed the data. XH, HC, and JH wrote the manuscript. All authors contributed to the article and approved the submitted version.

## Conflict of Interest

The authors declare that the research was conducted in the absence of any commercial or financial relationships that could be construed as a potential conflict of interest.

## Publisher’s Note

All claims expressed in this article are solely those of the authors and do not necessarily represent those of their affiliated organizations, or those of the publisher, the editors and the reviewers. Any product that may be evaluated in this article, or claim that may be made by its manufacturer, is not guaranteed or endorsed by the publisher.
